# Roxadustat has risks of reversible central hypothyroidism in patients undergoing hemodialysis: a single-center retrospective cohort study

**DOI:** 10.1080/0886022X.2024.2410375

**Published:** 2024-10-08

**Authors:** Emiko Otsuka, Mineaki Kitamura, Satoshi Funakoshi, Hiroshi Mukae, Tomoya Nishino

**Affiliations:** a Department of Nephrology, Nagasaki University Graduate School of Biomedical Sciences, Nagasaki, Japan; b Nagasaki Renal Center, Nagasaki, Japan; c Department of Respiratory Medicine, Nagasaki University Graduate School of Biomedical Sciences, Nagasaki, Japan

**Keywords:** Roxadustat, hypothyroidism, thyroid-stimulating hormone, hypoxia-inducible factor-prolyl hydroxylase inhibitor, hemodialysis

## Abstract

Roxadustat, a hypoxia-inducible factor-prolyl hydroxylase inhibitor, has proven efficacy in the treatment of renal anemia; however, evidence indicates that it may cause central hypothyroidism. The prevalence and reversibility of roxadustat-induced central hypothyroidism in patients undergoing hemodialysis remain unclear. Here, we retrospectively analyzed thyroid-stimulating hormone (TSH), free thyroxine (FT4), and free triiodothyronine (FT3) levels in 51 patients (mean age: 72.3 ± 10.7 years; 58.8% male) undergoing hemodialysis before, during, and after halting roxadustat treatment. TSH levels were significantly decreased from a median of 2.46 (interquartile range:1.60–4.51) mU/L before roxadustat treatment to 1.36 (0.72–2.41) mU/L during treatment (*p* < 0.001), and improved to 2.56 (1.78–4.63) mU/L after halting roxadustat (*p* < 0.001). Similarly, FT4 levels decreased from 1.11 (0.97–1.24) ng/dL before roxadustat treatment to 0.92 (0.71–1.03) ng/dL during treatment (*p* < 0.001) and improved to 1.05 (0.93–1.17) ng/dL after halting roxadustat (*p* < 0.001). FT3 levels were 2.04 (1.78–2.31) pg/mL before starting roxadustat, 1.97 (1.69–2.27) pg/mL during treatment, and 1.90 (1.63–2.18) pg/mL after halting roxadustat, with no significant difference between each group. Moreover, 2.0% of patients exhibited extremely low TSH levels (≤0.1 mU/L) and low TSH levels (>0.1 mU/L to <0.4 mU/L) before starting roxadustat and that percentage increased to 5.9% and 7.8%, respectively, during treatment. After roxadustat cessation, extremely low or low TSH levels recovered in all patients. Taken together, the results indicate that roxadustat can cause reversible central hypothyroidism in patients undergoing hemodialysis.

## Introduction

Anemia frequently occurs in patients with chronic kidney disease (CKD) [1], affecting over 90% of those undergoing hemodialysis [2]. It is also associated with increased morbidity, mortality, and cardiovascular disease [3,4]. The primary cause of anemia in CKD includes reduced erythropoietin production in the kidneys and alterations in iron metabolism. Therefore, the current treatments involve the use of erythropoiesis-stimulating agents (ESAs) in addition to iron supplementation [5,6]. Hypoxia-inducible factor-prolyl hydroxylase inhibitors (HIF-PHIs) are a novel class of drugs for treating renal anemia [7]. Roxadustat is one of the HIF-PHIs, which demonstrated efficacy for renal anemia [8–13]. Although it has not been reported during clinical trials, cases of central hypothyroidism have been reported following widespread use of roxadustat [14,15]. The cases were reversible and thyroid hormone levels normalized following roxadustat discontinuation [14,15]. However, the prevalence of hypothyroidism upon initiating roxadustat and its reversibility remains unclear. Therefore, we conducted a retrospective study of patients undergoing hemodialysis who were administered roxadustat. Specifically, we analyzed thyroid-stimulating hormone (TSH), free thyroxine (FT4), and free triiodothyronine (FT3) levels before, during, and after halting treatment with roxadustat.

## Methods

### Study design and patients

This retrospective study was conducted at a single center. The inclusion criteria included patients who were undergoing hemodialysis, being treated with roxadustat but later transitioned to ESA at the Nagasaki Renal Center from June to August 2023, and over 18 years old. Patients were excluded if they had undergone total thyroidectomy before the study period, had undergone surgery on the thyroid gland during the study period, or had received different doses of levothyroxine and had withdrawn their consent to the study. We analyzed TSH, FT4, and FT3 levels in three periods: before starting roxadustat, during treatment with roxadustat, and after discontinuation of roxadustat treatment. All patients underwent hemodialysis at least thrice a week, with dialysis modes being either in-center or home-based.

### Collection and analysis of data

Patient characteristics, including age, sex, dialysis duration, blood examinations, complications, and current medications, were obtained from medical records. Blood sampling was conducted at the beginning of hemodialysis sessions for all patients. Patients undergoing in-center hemodialysis were treated thrice a week, with blood sampling conducted at the beginning of the first session of the week, which was two days after the last session. Patients undergoing home hemodialysis were treated four or more times a week and received in-center hemodialysis twice a month. Blood samplings were conducted at the beginning of the sessions on in-center hemodialysis days. All blood examinations, including those for TSH, FT4, and FT3 levels, were performed at BML, Inc. (Tokyo, Japan). The levels of these hormones were measured using an electrochemiluminescence immunoassay.

### Reference values

We analyzed alterations in TSH, FT4, and FT3 levels before, during, and after halting roxadustat treatment. Moreover, we categorized TSH values into five grades: extremely low (≤0.1 mU/L), low (>0.1 mU/L to <0.4 mU/L), normal (≥0.4 mU/L to ≤4 mU/L), high (>4 mU/L to <10 mU/L), and extremely high (≥10 mU/L). Subsequently, we analyzed the distribution of patients across these categories before, during, and after halting roxadustat treatment. The TSH values were categorized based on guidelines provided by the European Thyroid Association, which defines the normal TSH level as (≥0.4 mU/L to ≤4 mU/L) [16]. Moreover, patients with TSH values ≤0.1 mU/L are associated with a higher risk of adverse health outcomes compared to those with TSH values between >0.1 mU/L to <0.4 mU/L [16]. Additionally, patients with TSH values ≥10 mU/L are at an elevated risk of cardiovascular events compared to those with normal TSH levels [17].

### Statistical analysis

Normally distributed data are presented as mean ± standard deviation (SD), and non-normally distributed data are presented as median (interquartile range [IQR]). The Wilcoxon signed-rank test was used to analyze the differences in the continuous variables, and the Fisher exact test was used to analyze the differences in the categorical variables. The Friedman test was used to analyze the differences in TSH, FT4, and FT3 levels before, during, and after halting roxadustat treatment. The Dunn–Bonferroni post-test was used to analyze the differences between the values for each period. Statistical significance was set at *p* < 0.05. Statistical analyses were performed using the JMP Pro 17 (SAS Institute Inc., Cary, NC, USA).

### Ethical statements

The study was approved by the Clinical Research Ethics Committee of the Nagasaki Renal Center (Nagasaki, Japan) (approval number: 23033) and conducted in accordance with the 1964 Declaration of Helsinki and its subsequent amendments. The requirement for informed consent was waived by the Clinical Research Ethics Committee owing to the retrospective nature of the study.

## Results

**Figure 1.**
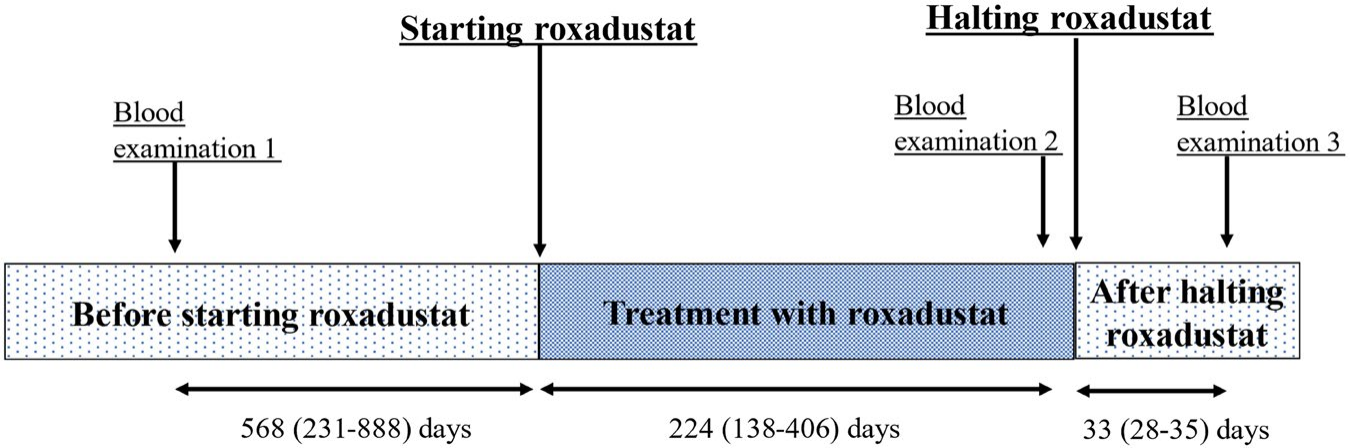
The duration of treatment with roxadustat and blood examinations. Blood examination 1. Blood samples before starting roxadustat. The median duration between blood examination 1 and starting roxadustat was 568 days (interquartile range [IQR]: 231–888). Blood examination 2. Blood samples from patients were treated with roxadustat. The median duration of roxadustat treatment until blood examination 2 was 224 days ([IQR]: 142–418). After the discontinuation of roxadustat, all patients were transitioned to erythropoietin stimulating agents (ESA) following blood examination 2. Blood examination 3. Blood examination after the discontinuation of roxadustat treatment. The median duration between the discontinuation of roxadustat and blood examination 3 was 33 days ([IQR]: 28–35).

Between July 2023 and August 2023, 52 patients undergoing hemodialysis were treated with roxadustat and underwent thyroid hormone evaluation at the Nagasaki Renal Center. One patient who had undergone total thyroidectomy was excluded. Finally, we analyzed the remaining 51 patients. Their mean age was 72.3 ± 10.7 years, and 58.8% of them were males. Of the 51 patients, seven were diagnosed with hypothyroidism before starting roxdustat and were prescribed levothyroxine at a daily dose of 25 (four patients), 50 (one patient), or 75 μg (two patients). The thyroid functions in these patients were well regulated, and the dosages of levothyroxine were not changed during the study period. Table 1 presents the baseline patient characteristics. The study participants were treated with a median roxadustat dose of 200 mg per week (IQR: 100–300 mg/week). After discontinuing roxadustat, their treatment was switched to an ESA (epoetin alfa, darbepoetin alfa, or epoetin β pegol). Blood examinations including those for TSH, FT4, and FT3 levels were conducted three times: blood test 1, before initiating roxadustat; blood test 2, during roxadustat treatment; blood test 3, after halting roxadustat treatment. The timing of blood tests and roxadustat initiation and discontinuation are shown in Figure 1 and Table 2.

**Table 1.** Baseline patient characteristics.

Characteristics	Total (*n* = 51)	Patients treated with levothyroxine (*n* = 7)	Patients not treated with levothyroxine (*n* = 44)	*p*-value
Age (years), *n* (%)	72.3 ± 10.7	73.5 ± 11.5	72.1 ± 10.7	0.85
Sex (male) (%)	30 (58.8%)	6 (85.7%)	24 (54.5%)	0.22
Dialysis vintage (years)	5.3 ± 5.5	6.6 ± 1.0	5.1 ± 5.9	0.008
Dialysis mode, *n* (%)				
In-center hemodialysis	47 (92.2%)	7 (100%)	40 (90.9%)	1.0
Home hemodialysis	4 (7.8%)	0 (0%)	4 (9.1%)	–
Dry weight (kg)	55.9 ± 12.5	50.4 ± 8.0	56.7 ± 13.0	0.16
Diabetes mellitus, *n* (%)	21 (41.2%)	4 (57.1%)	17 (38.6%)	0.36
Hypertension, *n* (%)	50 (98.0%)	7 (100%)	43 (97.7%)	0.69
Cardiovascular disease, *n* (%)	9 (17.6%)	2 (28.6%)	7 (15.9%)	0.42
Stroke, *n* (%)	10 (19.6%)	3 (42.9%)	7 (15.9%)	0.10
Hemoglobin (g/dL)	11.1 ± 1.2	10.5 ± 2.0	11.2 ± 1.0	0.70
Iron (μmol/L)	67.8 ± 35.6	84.9 ± 55.3	65.2 ± 31.2	0.46
Ferritin (ng/mL)	96.0 ± 100.4	118.2 ± 126.4	92.5 ± 96.9	0.78
TSAT (%)	23.5 ± 13.7	27.9 ± 17.9	22.8 ± 13.0	0.53
Creatinine (mg/dL)	8.8 ± 2.6	7.1 ± 2.4	9.1 ± 2.6	0.09
BUN (mg/dL)	56.1 ± 15.4	47.5 ± 17.4	57.5 ± 14.8	0.17
Total protein (g/dL)	6.4 ± 0.6	6.3 ± 0.9	6.5 ± 0.5	0.50
Albumin (g/dL)	3.3 ± 0.5	3.2 ± 0.8	3.3 ± 0.4	0.89
Potassium (mEq/L)	4.7 ± 0.7	4.9 ± 0.5	4.7 ± 0.7	0.29
Calcium (mg/dL)	8.8 ± 0.7	8.9 ± 0.6	8.8 ± 0.7	0.96
Phosphate (mg/dL)	5.0 ± 1.5	4.6 ± 1.5	5.1 ± 1.5	0.44
Total cholesterol (mg/dL)	115.5 ± 26.9	107.7 ± 32.3	118.0 ± 26.5	0.59
LDL cholesterol (mg/dL)	54.9 ± 19.1	44.4 ± 13.4	56.6 ± 19.5	0.15
Triglyceride (mg/dL)	86.7 ± 94.4	60.0 ± 17.8	91.0 ± 100.9	0.55
C-reactive protein (mg/dL)	0.4 ± 0.9	0.8 ± 1.5	0.3 ± 0.7	0.77
Doses of levothyroxine, *n* (%)				
None	44 (86.3%)	0 (0%)	44 (100%)	–
levothyroxine 25 μg	4 (7.8%)	4 (57.1%)	0 (0%)	–
levothyroxine 50 μg	1 (2.0%)	1 (14.3%)	0 (0%)	–
levothyroxine 75 μg	2 (3.9%)	2 (28.6%)	0 (0%)	–

The data are expressed as the mean ± standard deviation (SD). Sex (male), underlying complications, and levothyroxine doses are expressed as the number of patients and percentage. The Fisher exact test was used to analyze the differences in sex and dialysis mode between the patients treated with levothyroxine and those not treated with levothyroxine. The other differences were analyzed using the Wilcoxon signed-rank test.

TSAT: transferrin saturation; BUN: blood urea nitrogen; LDL cholesterol: low-density lipoprotein.

**Table 2.** Doses of roxadustat and durations of treatment and blood test.

	Total (*n* = 51)	Patients treated with levothyroxine (*n* = 7)	Patients not treated with levothyroxine (*n* = 44)	*p*-value
Doses of roxadustat (mg/week)	200 (100–300)	200 (100–300)	150 (100–300)	0.48
Durations between the blood test 1 and starting roxadustat (days)	568 (231–888)	161 (35–1172)	578 (338–883)	0.11
Durations between starting roxadustat and the blood test 2 (days)	224 (138–406)	404 (182–531)	224 (125–321)	0.17
Durations between halting roxadustat and blood test 3 (days)	33 (28–35)	33 (28–35)	33 (28–35)	0.97
Agents after transitioned, n (%)				
Epoetin alfa	38 (74.5%)	5 (71.4%)	33 (75.0%)	1.00
Darbepoetin	7 (13.7%)	2 (28.7%)	5 (11.4%)	0.24
Epoetin beta pegol	6 (11.7%)	0 (0%)	6 (13.6%)	0.58

The data are expressed as median (interquartile range). The number of patients and the percentage of each agent after transitioning are expressed as numbers (percentages). The Wilcoxon signed-rank test was used to analyze the differences in the dose of roxadustat and each duration between the patients treated with levothyroxine and those not treated with levothyroxine. The Fisher exact test was used to analyze the differences in the agents after transitioning.

**Figure 2.**
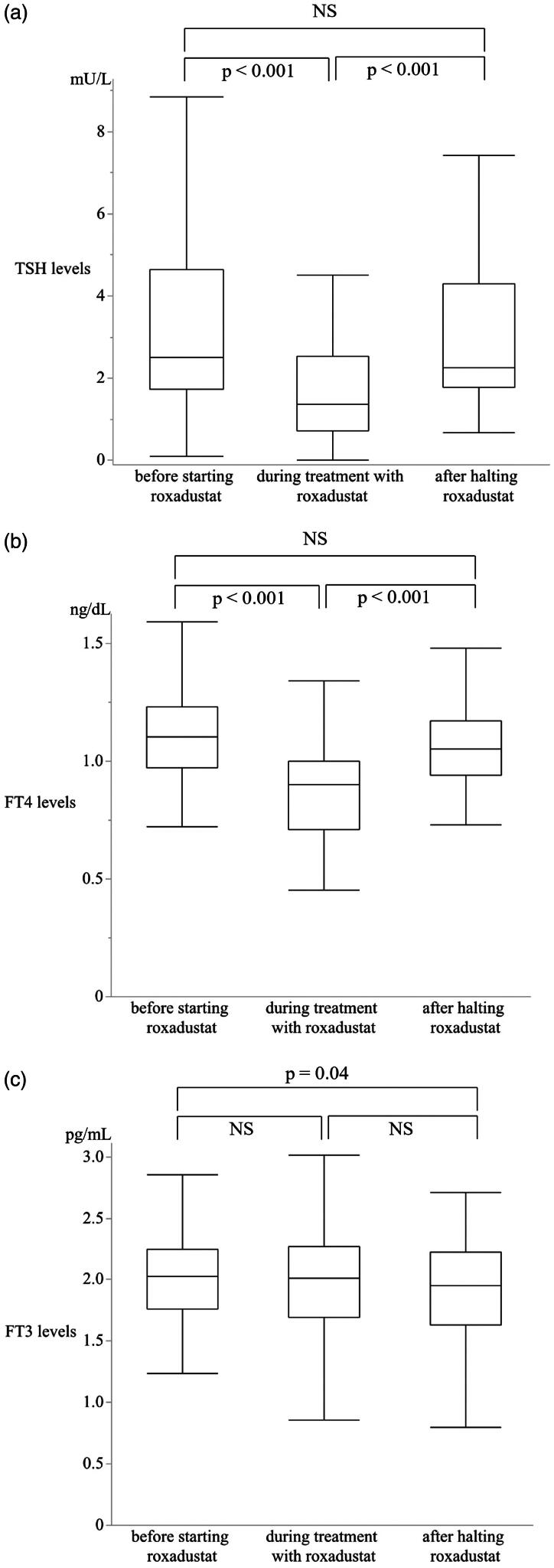
Alterations in thyroid stimulating hormone (TSH) (a), FT4 (b), and FT3 (c) levels during roxadustat treatment and after its discontinuation in all patients. Wilcoxon signed-rank test was used for the analysis.

The levels of TSH, FT4, and FT3 in the 51 patients before, during, and after halting roxadustat treatment are shown in Table 3. Compared to the levels before roxadustat initiation, TSH (*p* < 0.001) and FT4 (*p* < 0.001) levels were significantly reduced during treatment. On the contrary, after discontinuation of treatment, there were clear elevations in TSH (*p* < 0.001) and FT4 (*p* < 0.001) levels compared to the levels during treatment (Figure 2(a,b)). There was no significant change in FT3 levels in patients before and during treatment (*p* = 0.12), and during and after treatment (*p* = 0.08) (Figure 2(c)). The reduction in TSH levels during the treatment was also observed in seven patients treated with levothyroxine and in 44 patients not treated with levothyroxine (Supplementary Table 1 and Supplementary Figures 1 and 2).

**Table 3.** Levels of TSH, FT4, and FT3 before, during, and after treatment.

	Before starting roxadustat	During the treatment with roxadustat	After halting roxadustat	*p*-value
TSH (mU/L)	2.46 (1.60–4.51)	1.36 (0.72–2.41)	2.56 (1.78–4.63)	<0.001
FT4 (ng/dL)	1.11 (0.97–1.24)	0.92 (0.71–1.03)	1.05 (0.93–1.17)	<0.001
FT3 (pg/mL)	2.04 (1.78–2.31)	1.97 (1.69–2.27)	1.90 (1.63–2.18)	0.007

The data are presented as median (interquartile range). The Friedman test was used to analyze the differences in TSH, FT4, and FT3 levels in the three periods.

TSH, thyroid-stimulating hormone; FT4, free thyroxine; FT3, free triiodothyronine.

TSH levels during each period are shown in Table 4, and those with and without levothyroxine treatment are shown in Supplementary Table 2. The number of patients showing extremely low or low TSH levels was increased during roxadustat treatment; however, the TSH levels recovered in all these patients after halting roxadustat (*p* = 0.02). No correlation was noted between the dosage of roxadustat and negative feedback of TSH levels (data not shown).

**Table 4.** The number and percentage of patients for each TSH grade among the total number of patients.

TSH levels (mU/L)	Total (*n* = 51)		
	Before starting roxadustat	During treatment with roxadustat	After halting roxadustat
Extremely low (≤0.1)	1 (2.0%)	3 (5.9%)	0 (0%)
Low (>0.1, <0.4)	1 (2.0%)	4 (7.8%)	0 (0%)
Normal (≥0.4, ≤4)	35 (68.6%)	39 (76.4%)	37 (72.5%)
High (>4, <10)	13 (25.5%)	3 (5.9%)	11 (21.6%)
Extremely high (≥10)	1 (2.0%)	2 (3.9%)	3 (5.9%)

The TSH levels were divided into five grades; the number and percentage of patients in each group before starting roxadustat, undergoing treatment with roxadustat, and after halting roxadustat are shown. The Fisher exact test was used to analyze the differences; *p*-value = 0.02.

TSH, thyroid-stimulating hormone.

## Discussion

HIF-PHIs are a novel class of orally administered drugs that are used for the treatment of renal anemia. They activate the HIF oxygen-sensing pathway and effectively rectify and maintain hemoglobin levels in patients with CKD [18]. HIF comprises two subunits: an oxygen-sensitive α-subunit and a constitutively expressed β-subunit [18]. HIF-α and HIF-β are consistently synthesized regardless of oxygen concentration; however, HIF-α is degraded during normoxia [19,20]. Under hypoxic conditions or during HIF-PHI use, HIF-α accumulates in cells. Subsequently, HIF-α heterodimerizes with HIF-β and the transcription of HIF-regulated genes increases. This enhances anemia through increased endogenous erythropoietin (EPO) production and modulation of iron metabolism [18]. Roxadustat enhances hemoglobin levels in a manner similar to ESAs, without increasing cardiovascular events or compromising overall safety [21–24].

Following widespread roxadustat use, cases of central hypothyroidism have been reported [14,15]. TSH, FT3, and FT4 levels were reported to be significantly lower in the roxadustat group than those in the recombinant human erythropoietin (rHuEPO) group in a study of 110 patients with CKD, including those undergoing hemodialysis, peritoneal dialysis, and nondialysis (*p* < 0.05) [25]. A meta-analysis also showed that the incidence of suppression of thyroid function was significantly higher with roxadustat use than with ESA use (odds ratio: 6.45; 95% confidence interval: 3.39–12.27) [26]. Recently, Tanaka et al. reported 410 cases of hypothyroidism associated with roxadustat use recorded in the Japanese Adverse Drug Event Report (JADER) database between the inception of database in April 2004 and March 2023. The affected patients recovered in 42.9%, improved in 14.1%, and did not recover in 16.8% of the cases. The outcomes for the remaining 24.6% of patients were unavailable [27]. In addition, patients with asymptomatic hypothyroidism tend not to be reported because thyroid function tests are unlikely to be performed. Furthermore, patients with overt hypothyroidism can develop various symptoms [28]. In particular, patients undergoing hemodialysis have numerous underlying medical conditions that may hinder the recognition of hypothyroidism. In this study, none of the patients had obvious symptoms of hypothyroidism, although TSH and FT4 levels were significantly low in patients undergoing hemodialysis during roxadustat treatment (*p* < 0.001). Notably, hypothyroidism is associated with higher risks of cardiac mortality and all-cause mortality compared with euthyroidism and subclinical hypothyroidism [29]. On the contrary, Haraguchi et al. reported that roxadustat suppresses the levels of thyroid hormones, but might not lead to the development of hypothyroidism because it does not affect the lipid profile [30]. Roxadustat may suppress the hypothalamus–pituitary–thyroid axis but act as an agonist in other organs [31].

Moreover, after starting roxadustat, the percentage of patients exhibiting extremely low and low TSH levels increased from 2.0% to 5.9% and from 2.0% to 7.8%, respectively. After halting roxadustat, none of the patients showed extremely low or low TSH levels. Blood examinations after halting treatment were performed 33 days (IQR: 28–35) after the discontinuation of roxadustat treatment. Our results indicated that roxadustat-induced central hypothyroidism was reversible; however, this needs to be confirmed in extended follow-up blood examinations.

Unlike roxadustat, daprodustat does not suppress TSH levels [30,32]. This indicates that central hypothyroidism may not be associated with all HIF-PHIs but is rather specific to roxadustat. Thyroid hormones bind to thyroid hormone receptors (THR) and regulate various physiological and metabolic processes. THR comprises two distinct isoforms: THR-α and β [33]. THR-β plays crucial roles in biochemical processes, such as hepatic and kidney-related functions, cholesterol reduction, and hypothalamus-pituitary-thyroid regulation [33,34]. THR-β induces negative feedback in the hypothalamus-pituitary-thyroid axis by binding to the negative ligand of T3 [35]. Roxadustat, possessing a structure identical to the negative ligand of T3, can bind to THR-β, resulting in a negative feedback loop in the hypothalamus-pituitary-thyroid axis [35]. Furthermore, roxadustat can cross the blood-brain barrier in mice [36]. Through this mechanism, roxadustat can induce reversible central hypothyroidism. This is evidenced by previous case reports demonstrating enhanced TSH levels after the discontinuation of roxadustat treatment [14,15]. In contrast, patients with roxadustat-induced hypothyroidism do not typically show symptoms of hypothyroidism. Thyroid hormones stimulate THR-β in the liver, leading to increased cholesterol clearance and decreased blood cholesterol levels [37]. Thus, cholesterol levels increase during hypothyroidism. However, some reports indicate that patients with roxadustat-induced hypothyroidism exhibit decreased cholesterol levels, rather than increased levels [30,31,38]. Therefore, it is speculated that reduced cholesterol levels are a result of the agonistic effect of roxadustat on THRβ in the liver [31,38]. Although the effects of roxadustat in organs other than the liver remain unclear, we believe that the fact that roxadustat lowers TSH as well as cholesterol levels, supports the mechanism in which roxadustat mimics T3 and binds to THR, as shown by Yao et al. [35]. Whether the reduction in thyroid hormone levels caused by roxadustat is clinically significant hypothyroidism and whether discontinuation of roxadustat should be considered remain unclear. These issues need to be addressed in future studies.

This study has two strengths. First, all participants were patients undergoing hemodialysis. Second, TSH, FT4, and FT3 levels were assessed in all patients before, during, and after halting treatment.

This study has some limitations. First, as this was a retrospective study, the timing of blood sampling varied among cases; before initiating roxadustat, during roxadustat treatment, and after discontinuation of roxadustat. In particular, the thyroid hormone levels before initiating roxadustat might be slightly different from those immediately before initiation, although the thyroid functions were well regulated before initiating roxadustat. As this was a retrospective study, we did not know the association between roxadustat and hypothyroidism at the time of blood test 1. Therefore, the timing of blood tests varied depending on the case. Second, we could not investigate the underlying medical conditions of patients treated with levothyroxine. Third, we did not conduct repetitive thyroid hormone level assessments after roxadustat discontinuation. Fourth, the accurate timing of reduction in the thyroid hormone levels could not be clarified, as this study was conducted at a single center and the sample size was small.

In conclusion, roxadustat reversibly suppressed TSH levels in patients undergoing hemodialysis. TSH levels recovered approximately 4–5 weeks after the discontinuation of roxadustat treatment. Patients with low TSH levels may be asymptomatic and clinicians should be vigilant to changes in thyroid hormone levels, with or without symptoms, during roxadustat treatment.

## Supplementary Material

Supplementary Figure 1a.pdf

Supplementary Table 2 0901.docx

Supplementary Figure 1b.pdf

Supplementary Figure 2b.pdf

Supplementary Figure 2a.pdf

Supplementary Figure 2c.pdf

Supplementary Figure 1c.pdf

Supplementary Table 1 0901.docx
