# Supplementary figures and images for: Roxadustat has risks of reversible central hypothyroidism in patients undergoing hemodialysis: a single-center retrospective cohort study
Source: Ren Fail. 2024 Oct 8;46(2):2410375. doi: 10.1080/0886022X.2024.2410375 (PMC11463015; doi:10.1080/0886022X.2024.2410375)

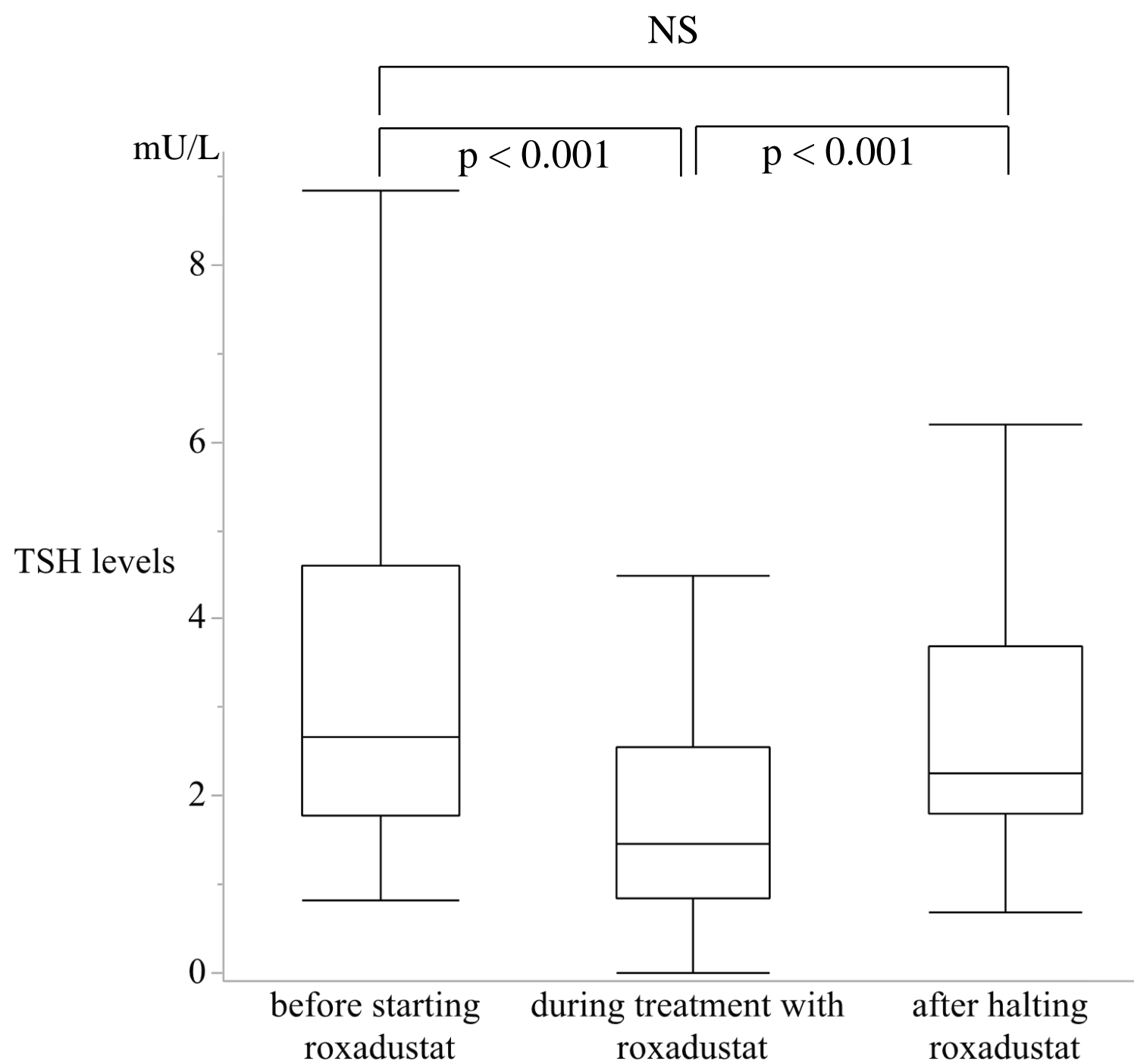

Supplementary Figure 1a

Supplement: Supplementary Figure 1a.pdf [file IRNF_A_2410375_SM2003.pdf]

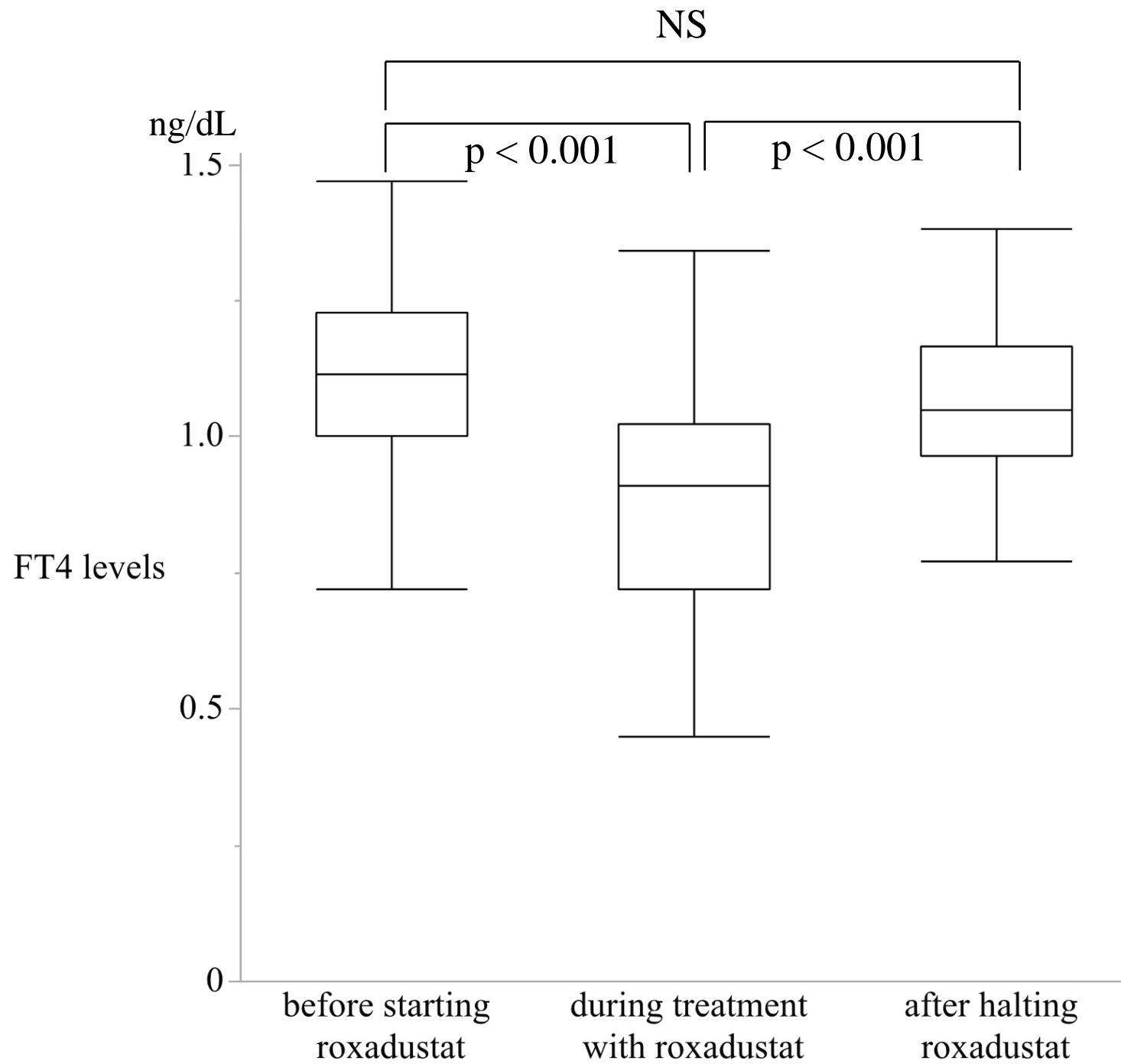

Supplementary Figure 1b

Supplement: Supplementary Figure 1b.pdf [file IRNF_A_2410375_SM2001.pdf]

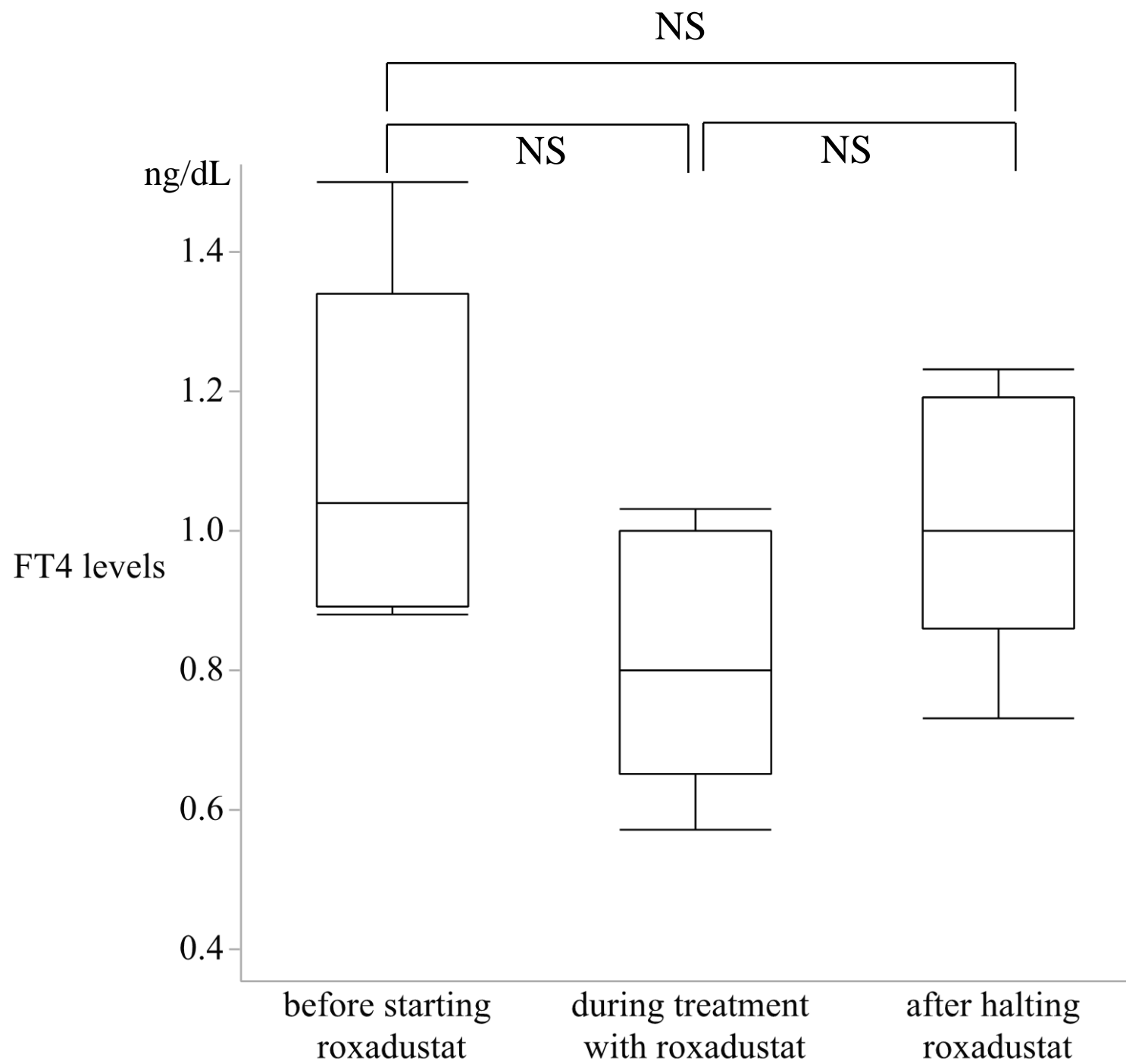

Supplementary Figure 2b

Supplement: Supplementary Figure 2b.pdf [file IRNF_A_2410375_SM2000.pdf]

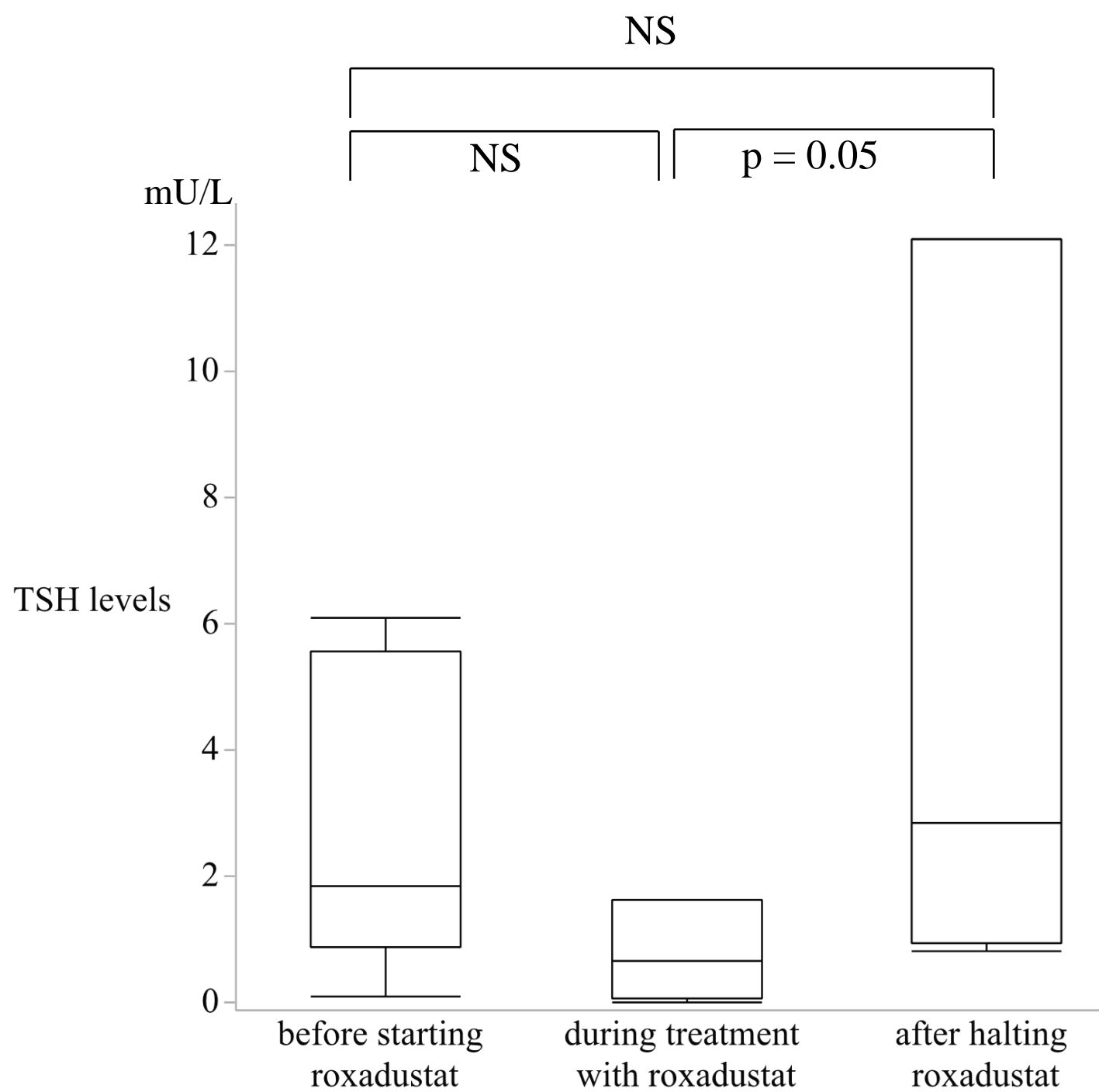

Supplementary Figure 2a

Supplement: Supplementary Figure 2a.pdf [file IRNF_A_2410375_SM1999.pdf]

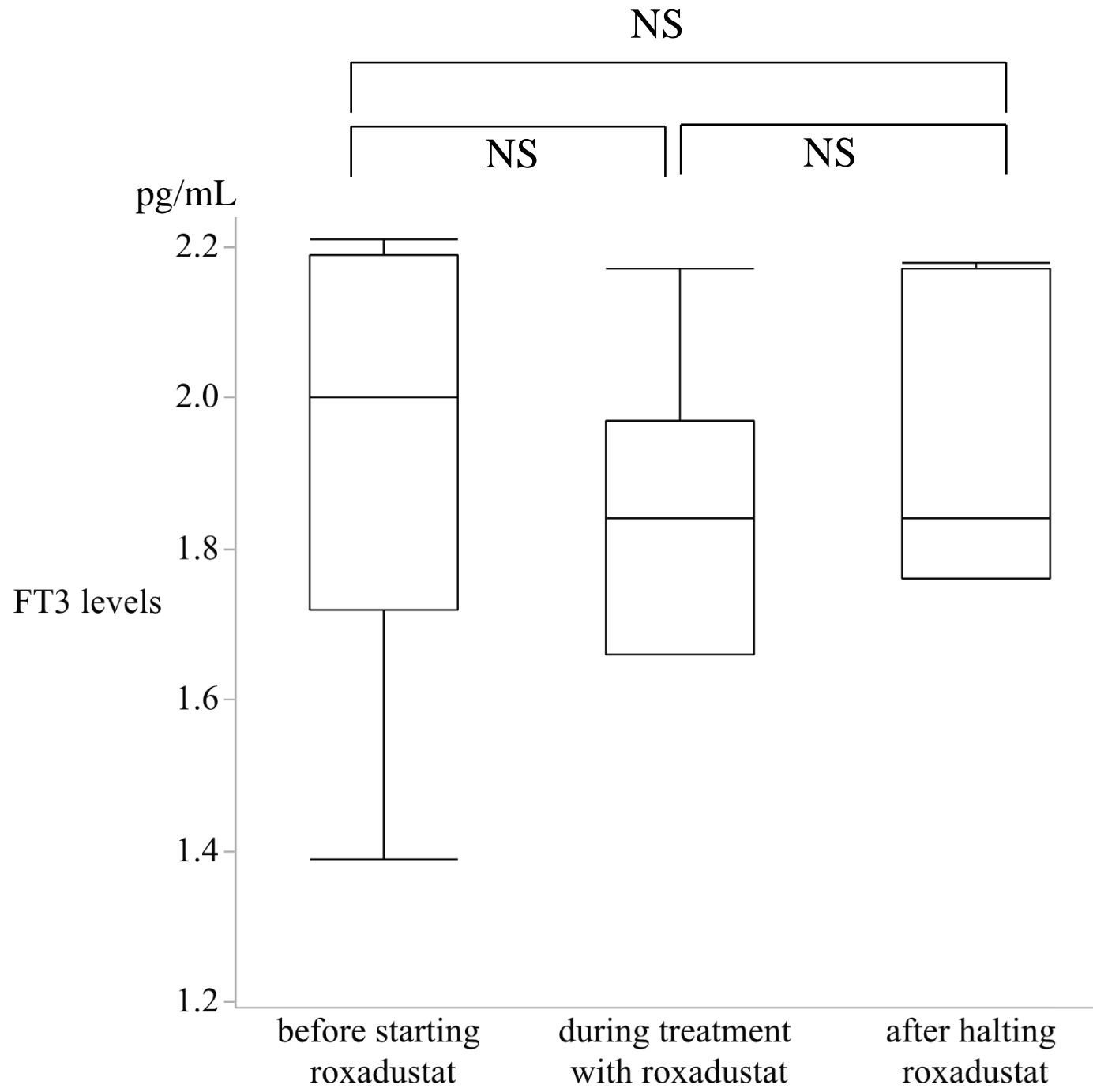

Supplementary Figure 2c

Supplement: Supplementary Figure 2c.pdf [file IRNF_A_2410375_SM1998.pdf]

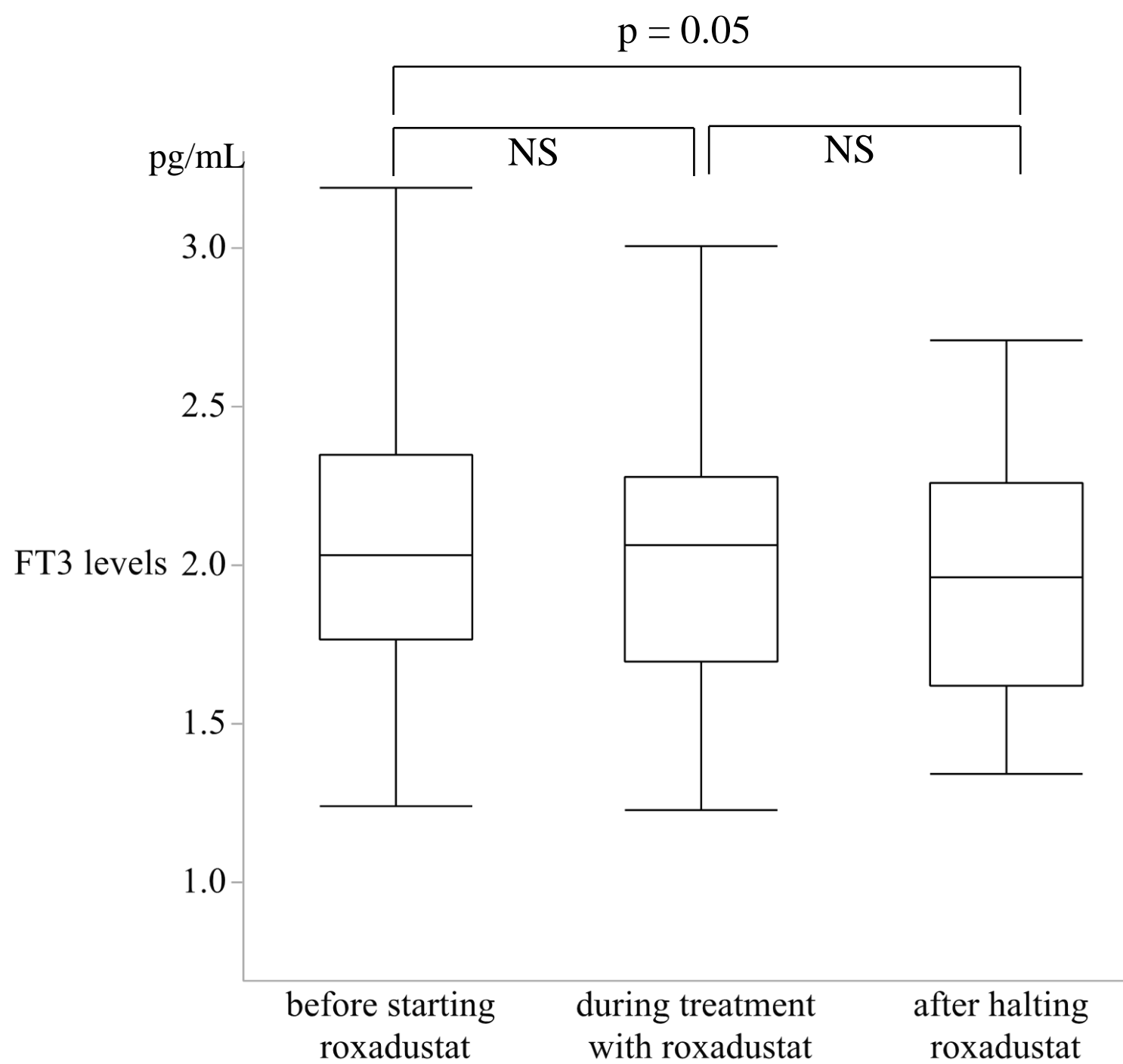

Supplementary Figure 1c

Supplement: Supplementary Figure 1c.pdf [file IRNF_A_2410375_SM1997.pdf]
