# Supplementary material for: Roxadustat has risks of reversible central hypothyroidism in patients undergoing hemodialysis: a single-center retrospective cohort study
Source: Ren Fail. 2024 Oct 8;46(2):2410375. doi: 10.1080/0886022X.2024.2410375 (PMC11463015; doi:10.1080/0886022X.2024.2410375)
Supplement: Supplementary Table 2 0901.docx [file IRNF_A_2410375_SM2002.docx]

**Supplementary Table 2. The number and percentages of each TSH grade in patients treated and not treated with levothyroxine**

| TSH levels (mU/L) | With levothyroxine (n=7) | | | Without levothyroxine (n=44) | | |
| --- | --- | --- | --- | --- | --- | --- |
|  | Before starting roxadustat | During treatment with roxadustat | After halting roxadustat | Before starting roxadustat | During treatment with roxadustat | After halting roxadustat |
| Extremely low (0.1≦) | 0  (0%) | 0  (0%) | 0  (0%) | 1  (2.2%) | 3  (6.8%) | 0  (0%) |
| Low (0.1<, <0.4) | 0  (0%) | 1  (14.3%) | 0  (0%) | 1  (2.2%) | 3  (6.8%) | 0  (0%) |
| Normal (0.4≦, ≦4) | 0  (0%) | 4  (57.1%) | 1  (14.3%) | 35  (79.5%) | 35  (79.5%) | 36  (81.8%) |
| High (4<, <10) | 6  (85.7%) | 0  (0%) | 3  (42.9%) | 7  (15.9%) | 3  (6.8%) | 8  (18.2%) |
| Extremely  high (10≦) | 1  (14.3%) | 2  (28.6%) | 3  (42.9%) | 0  (0%) | 0  (0%) | 0  (0%) |

The TSH levels were divided into five grades, with the number and percentage of patients before starting roxadustat, undergoing treatment with roxadustat, and after halting roxadustat in patients with and without levothyroxine.

TSH, thyroid-stimulating hormone.
