# Supplementary material for: Roxadustat has risks of reversible central hypothyroidism in patients undergoing hemodialysis: a single-center retrospective cohort study
Source: Ren Fail. 2024 Oct 8;46(2):2410375. doi: 10.1080/0886022X.2024.2410375 (PMC11463015; doi:10.1080/0886022X.2024.2410375)
Supplement: Supplementary Table 1 0901.docx [file IRNF_A_2410375_SM1996.docx]

**Supplementary Table 1. TSH, FT4, and FT3 levels before, during, and after treatment in patients treated with and not treated with levothyroxine**

|  | Before starting roxadustat | During the treatment with roxadustat | After halting roxadustat | p value |
| --- | --- | --- | --- | --- |
| With levothyroxine | |  |  |  |
| TSH (mU/L) | 7.88 (5.89–9.57) | 1.65 (1.36–11.51) | 6.19 (4.30–28.08) | 0.050 |
| FT4 (ng/dL) | 1.06 (0.92–1.09) | 0.71 (0.65–0.78) | 0.86 (0.73–0.93) | 0.004 |
| FT3 (pg/mL) | 1.72 (1.53–2.21) | 2.06 (1.33–2.22) | 1.88 (1.65–2.07) | 0.005 |
| Without levothyroxine |  |  |  |  |
| TSH (mU/L) | 2.19 (1.42–3.40) | 1.35 (0.69–2.08) | 2.15 (1.67–3.40) | < 0.001 |
| FT4 (ng/dL) | 1.14 (1.00–1.27) | 0.95 (0.78–1.03) | 1.05 (0.99–1.19) | < 0.001 |
| FT3 (pg/mL) | 2.05 (1.81–2.35) | 1.96 (1.69–2.27) | 1.95 (1.62–2.25) | NS |

The data are presented as median (interquartile range), and the Friedman test was used to analyze the differences in TSH, FT4, and FT3 levels in three periods.

TSH, thyroid-stimulating hormone; FT4, free thyroxine; FT3, free triiodothyronine.
